# pPCV, a versatile vector for cloning PCR products

**DOI:** 10.1186/2193-1801-2-441

**Published:** 2013-09-05

**Authors:** Christiane R Janner, Ana Lívia P Brito, Lidia Maria P Moraes, Viviane CB Reis, Fernando AG Torres

**Affiliations:** Centro de Biotecnologia Molecular, Departamento de Biologia Celular, Universidade de Brasília, Brasília, DF, 70910-900 Brazil

**Keywords:** Polymerase chain reaction, Molecular cloning, Plasmid

## Abstract

The efficiency of PCR product cloning depends on the nature of the DNA polymerase employed because amplicons may have blunt-ends or 3′ adenosines overhangs. Therefore, for amplicon cloning, available commercial vectors are either blunt-ended or have a single 3′ overhanging thymidine. The aim of this work was to offer in a single vector the ability to clone both types of PCR products. For that purpose, a minimal polylinker was designed to include restriction sites for *Eco*RV and *Xcm*I which enable direct cloning of amplicons bearing blunt-ends or A-overhangs, respectively, still offering blue/white selection. When tested, the resulting vector, pPCV, presented high efficiency cloning of both types of amplicons.

## Introduction

The *in vitro* amplification of DNA fragments by polymerase chain reaction (PCR) is a routine technique in most molecular biology laboratories. Direct cloning of DNA fragments amplified by *Taq* DNA polymerase has frequently been found to be inefficient [Harrison et al. 1994] since this enzyme tends to add a non-templated nucleotide to the 3′ ends of the amplicon, mostly an adenosine residue, leaving a 3′overhang [Clark ] since this enzyme tends to add a non-templated nucleotide to the 3′ ends of the amplicon, mostly an adenosine residue, leaving a 3′overhang [Clark ] since this enzyme tends to add a non-templated nucleotide to the 3′ ends of the amplicon, mostly an adenosine residue, leaving a 3′overhang [Clark 1988. To circumvent this limitation, some commercially available vectors were constructed in order to have a 3′-T overhang (T-vectors) for sticky-end cloning. Many strategies have been developed to add a 3′-T overhang. One approach involves tailing a blunt-ended vector using terminal transferase in the presence of dideoxythymidine triphosphate (ddTTP) [Holton & Graham 1991 but there is a high probability that some vector molecules will lack an overhang at one or both ends. These incomplete plasmids can circularize during ligation rendering ineffective for cloning [Jun et al. 2010. Another approach is to digest a parental vector with a restriction enzyme that will generate single 3′-T overhangs. Restriction enzymes used for that purpose include *Bci*VI, *Bfi*I, *Hph*I, *Mnl*I, *Taa*I, *Xcm*I and *Eam*1105I [Jun et al. 2010; Dimov 2012; Gu & Ye 2011; Borovkov & Rivkin 1997. However, these vectors are not recommended for cloning amplicons produced by DNA polymerases which generate blunt-ended products.

The aim of this work was to construct a vector based on pBlueScript® II KS with a modified polylinker which would allow direct cloning of PCR products bearing either blunt-ends or A-overhangs.

## Materials and methods

### Strain and media

*Escherichia coli* XL10-Gold and DH5α were used for routine DNA manipulations. Bacterial cells were cultured in LB medium (0.5% yeast extract, 1% peptone and 1% NaCl) supplied with 100 μg/ml of ampicillin, 0.1 mM IPTG and 0.004% X-Gal (5-bromo-4-chloro-3-indolyl-β-D-galactopyranoside) when necessary. Genomic DNA of *Saccharomyces cerevisiae* S288c (*MATα SUC2 mal gal2 mel flo1 flo8-1 hap1 ho bio1 bio6*) [Mortimer & Johnston 1986 was used as template for amplification of the *LEU2* gene.

### Construction of T-vector

The stuffer DNA used in this work was derived from a fragment of the *S. cerevisiae URA*3 gene present in plasmid pNKY51 [Alani et al. 1987 and was obtained by PCR using the following primers: PXCM-1 (5′-AAGGTACCGATATCTCCAATACTTGT*ATGGAGGGCACAGTTAAGCC*-3′) and PXCM2 (5′-AAGAGCTCGATATCCTCCAATACTCCTTTGG*ATCCCTTCCCTTTGCAAATAGT*-3′). Primer PXCM-1 contains restriction sites for *Sac*I, *Eco*RV and *Xcm*I while PXCM-2 has sites for *Kpn*I, *Eco*RV and *Xcm*I (all sites are underlined). Both primers have sequences complementary to *URA3* which allow amplification of a ~600 pb stuffer DNA fragment. PCR was carried out in a volume of 50 μL containing 1.5 ng pNKY51, 0.2 mM dNTP, 0.2 μM each primer, 1× PCR buffer (100 mM Tris–HCl [pH 8.5], 500 mM KCl), 2 mM MgCl_2_ and 2 U *Taq* polymerase (LCG Biotechnology). Amplification was performed for 30 cycles of 94°C/45 s, 65°C/45 s, 72°C/40 s after an initial denaturation step of 94°C/45 s. A final extension step was performed for 2 min/72°C. The resulting amplicon was purified with UltraClean PCR Clean-Up Kit (MO BIO) and digested with *Sac*I and *Kpn*I following ligation to pBlueScript® II KS digested with the same enzymes.

### Cloning efficiency

To test cloning efficiency of both vectors, the *S. cerevisiae LEU2* gene was cloned after amplification from yeast genomic DNA using *Taq* polymerase (Invitrogen) or *Phusion* (Finnzymes) and primers 5-leud (5′-GAGATCTATATATATTTCAAGGATATACCATTCTAATG-3′) and 3-leud (5′-GAGATCTGTTTCATGATTTTCTGTTACACC-3′). Both amplification reactions were carried out in a volume of 50 μL. For amplification with *Taq* polymerase, 10 ng genomic DNA was added to a reaction which included 1× PCR buffer (200 mM Tris–HCl [pH 8.4], 500 mM KCl), 2 mM MgCl_2_, 0.2 mM dNTP mixture, 0.2 μM each primer and 2 U *Taq* polymerase. The reaction was performed for 30 cycles of 94°C/45 s, 55°C/30 s, 72°C/1.5 min after an initial denaturation of 94°C/45 s. The final extension was accomplished for 10 min/72°C. The PCR system with *Phusion* was carried out with 10 ng genomic DNA, 1× *Phusion* HF buffer (1.5 mM MgCl_2_), 0.2 mM dNTP, 0.5 μM each primer and 0.5 U *Phusion* DNA polymerase. The PCR program was: 30 s at 98°C for initial denaturation following 30 cycles of 98°C/10 s, 61°C/30 s, 72°C/30 s with a final extension of 72°C/5 min. PCR products were purified as described previously and ligated into the constructed cloning vectors. Ligation was carried out in a final volume of 10 μL with a vector:insert ratio of 1:5. The system included 1 U of T4 DNA ligase (USB) and 1× reaction buffer (66 mM Tris–HCl [pH 7.6], 6.6 mM MgCl_2_, 10 mM DTT, 66 μM ATP), and incubation was carried out at 16°C for 16 h following transformation of *E. coli* DH5α cells.

## Results and discussion

**Figure 1:**
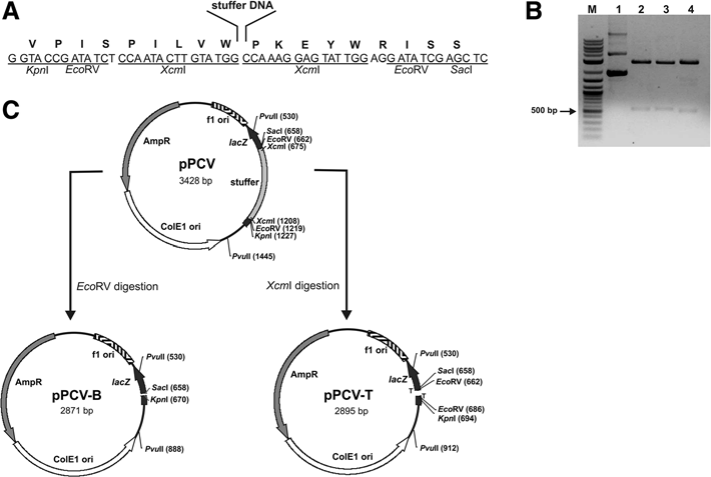
**Construction of pPCV. (A)** Restriction sites and predicted *lacZα* reading frame present in pPCV **(B)** Restriction analysis of pPCV. Lanes: M-2log molecular weight marker (New England Biolabs); 1-intact pPCV; 2-pPCV digested with *Eco*RV; 3-pPCV digested with *Sac*I and *Kpn*I; 4-pPCV digested with *Xcm*I. **(C)** Physical map of pPCV and its linearized forms pPCV-T and pPCV-B.

For vector construction, a minimal polylinker was designed (Figure 1A) with the inclusion of restriction sites for *Xcm*I, which produce 3′-T overhangs that can be used for cloning PCR products derived from amplification by *Taq* polymerase, and *Eco*RV, which yields blunt-ends suitable for cloning PCR products generated by *Pfu* DNA polymerases. It is argued that the use of *Xcm*I is limited because vectors incubated with this enzyme are often partially digested leading to a high background of non-recombinant transformants [Xuejun et al. 2002]. This issue was solved by the insertion of a stuffer DNA sequence large enough to be easily separated by gel electrophoresis [Gu & Ye 2011; Jo & Jo 2001. The new polylinker still allows blue/white selection because the *lacZα* reading frame is reestablished upon religation of the vector after removal of the stuffer DNA (Figure 1A). When vectors digested with *Eco*RV are religated the *lacZα* reading frame is restored thus rendering the cells blue, whereas vectors digested with *Xcm*I can only yield blue colonies if both T-overhangs are lost prior to religation.

For stuffer DNA, a fragment of the yeast *URA3* gene was amplified containing *Eco*RV and *Xcm*I sites for amplicon ligation and *Sac*I and *Kpn*I for cloning into pBlueScript® II KS digested with the same enzymes (Figure 1A). A selected clone was digested with different enzymes to confirm the presence of the stuffer DNA: *Eco*RV (558 bp), *Sac*I + *Kpn*I (570 bp), *Xcm*I (534 bp) (Figure 1B). The resulting vector was named pPCV (Figure 1C). This vector was digested either with *Xcm*I or *Eco*RV and the ~2.9 kb versions of the linearized vectors were named pPCV-T and pPCV-B, respectively (Figure 1C).

**Figure 2:**
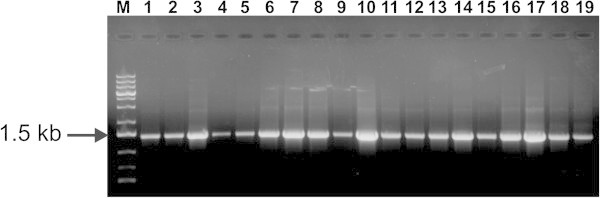
**Confirmation of the presence of inserts.** PCR was carried out using as template plasmid DNA isolated from randomly selected colonies derived from the pPCV-B (1-10) or pPCV-T (11-19) ligation systems. M–GeneRuler 1 kb plus (Fermentas).

To test the efficiency of the resulting vectors, a yeast *LEU2* gene fragment was amplified by using *Phusion* or *Taq* polymerase and the resulting amplicons ( ~1.4 kb) were ligated into pPCV-B and pPCV-T, respectively. The results of bacterial transformation are presented on Table 1 and the presence of inserts was assessed by PCR using primers 5-leud and 3-leud (Figure 2). The low percentage of white colonies observed when the pPCV-B system was used is explained by the fact that ligation of blunt-ended molecules is generally more difficult than sticky-ends. Nonetheless, a high percentage (83.3%) of white colonies had inserts. As for the pPCV-T system, most of the white colonies (90.0%) observed had inserts. All other false positives can be explained by the loss of one T-overhang following religation, which results in the loss of original *lacZα* reading frame as has been previously observed [Arashi-Heese et al. 1999.

**Table 1:** **Cloning efficiency of pPCV**

System	% White colonies	% Recombinant clones
pPCV-B	4.7%	83.3%
pPCV-T	92.2%	90.0%

The results shown in this work show that pPCV can be successfully used for high efficiency cloning of amplicons. It provides in the same cloning platform two important advantages: i) the ability to clone PCR products derived from different DNA polymerases still allowing blue/white selection and, ii) its minimal polylinker prevents undesirable restriction sites at the ends of cloned amplicon after subcloning. Plasmid pPCV is available upon request.
